# The efficacy and safety of Sanqi Qushi Granule in patients with idiopathic membranous nephropathy ——protocol of a multicenter, randomized control trial (SQ-AUTUMN)

**DOI:** 10.1186/s12906-023-03950-9

**Published:** 2023-04-27

**Authors:** Chuang Li, Wenjun Shan, Xing Liang, Qinghua Zhang, Xindong Qin, Sha Jiang, Xiaofan Hong, Lijuan Wang, Ping Li, Haowen Gu, Yi Wang, Kun Bao

**Affiliations:** 1 State Key Laboratory of Dampness Syndrome of Chinese Medicine, The Second Affiliated Hospital of Guangzhou University of Chinese Medicine, Guangzhou, China; 2 Guangdong-Hong Kong-Macau Joint Lab On Chinese Medicine and Immune Disease Research, Guangzhou, China; 3 Guangdong Provincial Key Laboratory of Chinese Medicine for Prevention and Treatment of Refractory Chronic Disease, The Second Affiliated Hospital of Guangzhou University of Chinese Medicine, Guangzhou, China; 4 Nephrology Department, Guangdong Provincial Hospital of Chinese Medicine, Guangzhou, China; 5 The Second Clinical College of Guangzhou University of Chinese Medicine, Guangzhou, China

**Keywords:** Sanqi Qushi Granule, Modified Ponticelli Regimen, Cyclophosphamide, Steroid, Idiopathic membranous nephropathy, Randomized controlled trial

## Abstract

**Background:**

Adult nephropathy is mainly caused by idiopathic membranous nephropathy (IMN). In cases of proteinuria, Modified Ponticelli Regimens (MPR) are often successful. However, it can cause adverse side effects. Oral Sanqi Qushi Granule (SQG) with MPR is effective in patients with IMN. However, whether it can improve the remission rate of IMN and shorten the remission time is unknown. In this trial, SQG with MPR on IMN will be evaluated clinically for its efficacy and safety.

**Methods:**

We will randomly assign IMN patients who meet the criteria to receives SQG plus cyclical Cyclophosphamide (CTX)/steroids or with placebo plus cyclical CTX/steroids for 6 months. A 12-month follow-up will be conducted on them. Status of remission will be used to assess treatment efficacy.

**Discussion:**

This study aims to appraise whether treatment with SQG plus cyclical CTX/steroids is superior to placebo plus cyclical CTX/steroids in the remission rate of patients with adult IMN. Adverse events of SQG plus MPR will be also evaluated for further researches about Chinese Medicine and MPR on whether it can improve the remission rate of IMN in half a year and shorten the remission time and relieve adverse effects will also be clarified.

**Trial registration:**

Chinese Clinical Trial Registry ChiCTR2200061953. Registered on 13 July 2022.

## Administrative information

Note: the numbers in curly brackets in this protocol refer to SPIRIT checklist item numbers. The order of the items has been modified to group similar items (see http://www.equator-network.org/reporting-guidelines/spirit-2013-statement-defining-standard-protocol-items-for-clinical-trials/).

**Table:** 

Title {1}	The efficacy and safety of Sanqi Qushi Granule in patients with idiopathic membranous nephropathy ——protocol of a multicenter, randomized control trial (SQ-AUTUMN)
Trial registration {2a and 2b}.	Chinese Clinical Trial Registry ChiCTR2200061953. Registered on 13 July 2022.
Protocol version {3}	Version 1.0 of 11–2021
Funding {4}	the Special Project of State Key Laboratory of Dampness Syndrome of Chinese Medicine (Nos. SZ2021ZZ36 and SZ2021ZZ09); the National Natural Science Foundation of China (No. 81974565); the 2020 Guangdong Provincial Science and Technology Innovation Strategy Special Fund (Guangdong-Hong Kong-Macau Joint Lab) (No. 2020B1212030006); the Natural Science Foundation of Guangdong Province (No.2022A1515011628); the Guangzhou Science and Technology Plan Project (No.202102010212).
Author details {5a}	Chuang Li:State Key Laboratory of Dampness Syndrome of Chinese Medicine, The Second Affiliated Hospital of Guangzhou University of Chinese Medicine, Guangzhou, China;Guangdong-Hong Kong-Macau Joint Lab on Chinese Medicine and Immune Disease Research, Guangzhou, China;Guangdong Provincial Key Laboratory of Chinese Medicine for Prevention and Treatment of Refractory Chronic Disease, The Second Affiliated Hospital of Guangzhou University of Chinese Medicine, Guangzhou, China;Nephrology Department,Guangdong Provincial Hospital of Chinese Medicine, Guangzhou, China. Wenjun Shan:The Second Clinical College of Guangzhou University of Chinese Medicine,Guangzhou, China. Xing Liang:Nephrology Department,Guangdong Provincial Hospital of Chinese Medicine, Guangzhou, China. Qinghua Zhang:Nephrology Department,Guangdong Provincial Hospital of Chinese Medicine, Guangzhou, China. Xindong Qin:Nephrology Department,Guangdong Provincial Hospital of Chinese Medicine, Guangzhou, China. Sha Jiang:Nephrology Department,Guangdong Provincial Hospital of Chinese Medicine, Guangzhou, China. Xiaofan Hong:Nephrology Department,Guangdong Provincial Hospital of Chinese Medicine, Guangzhou, China. Lijuan Wang:The Second Clinical College of Guangzhou University of Chinese Medicine,Guangzhou, China Ping Li:State Key Laboratory of Dampness Syndrome of Chinese Medicine, The Second Affiliated Hospital of Guangzhou University of Chinese Medicine, Guangzhou, China;Nephrology Department,Guangdong Provincial Hospital of Chinese Medicine, Guangzhou, China. Haowen Gu:The Second Clinical College of Guangzhou University of Chinese Medicine,Guangzhou, China. Yi Wang:The Second Clinical College of Guangzhou University of Chinese Medicine,Guangzhou, China. Kun Bao:State Key Laboratory of Dampness Syndrome of Chinese Medicine, The Second Affiliated Hospital of Guangzhou University of Chinese Medicine, Guangzhou, China;Guangdong-Hong Kong-Macau Joint Lab on Chinese Medicine and Immune Disease Research, Guangzhou, China;Guangdong Provincial Key Laboratory of Chinese Medicine for Prevention and Treatment of Refractory Chronic Disease, The Second Affiliated Hospital of Guangzhou University of Chinese Medicine, Guangzhou, China;Nephrology Department,Guangdong Provincial Hospital of Chinese Medicine, Guangzhou, China. KB and CL conceived and designed this study. WS and CL wrote the manuscript with contributions from all authors. XL, QZ, XQ, SJ, and XH assisted in revisions. LW,PL, HG and YW refined the protocol. All authors were instrumental in the completion of the article and reviewed the final version of the article.
Name and contact information for the trial sponsor {5b}	Kun Bao, baokun@aliyun.com
Role of sponsor {5c}	KB is the project leader. He conceived and designed this study.

## Introduction

### Background and rationale {6a}

In adults, idiopathic membranous nephropathy (IMN) is one of the more common causes of unexplained nephrotic syndrome [1]. It is estimated that IMN occurs in about 8–10 people per million annually [2] with a 2:1 predominance of male [3]. The relative frequency of IMN comprises approximately 14.3% of primary glomerular diseases [4] and 20.7%of the nephrotic syndrome in China [5]. IMN is a glomerular lesion caused by kidney-specific autoimmunity and is characterized by increased proteinuria which is responsible to the deposition of antibodies (usually IgG4) located in the subepithelial area of the glomerular basement membrane. Accumulative evidence reveals that in 70%–80% of patients, the antibodies are directed against M-type phospholipase A2 receptor(PLA2R) [6, 7]. Some patients with IMN are benign or indolent [8]. However, 30%-40% of patients develop end-stage renal disease (ESRD) within 5 -15 years [9].

The 2012 and 2021 Kidney Disease Improving Global Outcomes (KDIGO) guidelines proposed that steroids in combination with alkylating agents constituting Modified Ponticelli Regimen are applied to patients who expose to a high risk of developing ESRD [10]. In the KDIGO guidelines,cyclical Cyclophosphamide (CTX)/steroids therapy is recommended as the initial therapy. And it is preferably used treatment option in clinical practice. However, cyclical CTX/steroids use can cause adverse side effects, such as central obesity, infectious episodes, femoral head necrosis, and liver injury [11], and may increase the risk of cancer [12]. Its adverse reactions are serious, so it is greatly restricted in clinical practice.

Many people suffering from IMN seek help from adjuvant therapy. In China, many physicians are committed to treat IMN with Traditional Chinese Medicine (TCM) [13]. TCM is beneficial to relieve proteinuria, eliminate edema, and reduce complications of IMN [14]. Although several scattered clinical trials are reported, due to the lack of sufficient literature and methodological deficiencies, there is no exact conclusion on the efficacy of TCM on IMN.

Sanqi Qushi Granules (SQG) is one of formula to treat IMN which made by Guangdong Province Traditional Chinese Medical Hospital. It contains Tu Fu Ling (Rhizoma Smilacis Glabrae), Bai Zhu (Atractylodes macrocephala Koidz.), Huang Qi (Astragali Radix), Chan Hua (Isaria cicadae Miquel), E Zhu (Curcuma phaeocaulis Valeton),Chi Shao (Paeoniae Radix Rubra), San Qi (Panax notoginseng (Burkill) F. H. Chen ex C. H.). It is derived from Sanqi formula which has been utilized in the clinic for over 20 years with good efficiency in kidney disease [15, 16]. After a treatment period of 6 months for SQG plus cyclical CTX/steroids and basic treatment therapy, IMN patients with mediate-risk and high-risk factors achieved 78.3% clinical remission rate without apparent side effects in our previous study. However, there is no head-to-head comparison in a randomized controlled trial between SQG plus cyclical CTX/ steroids and cyclical CTX/ steroids.

For this, we planned a multicenter, randomized, double-blind, placebo-controlled trial. The clinical efficacy and safety of SQG plus cyclical CTX/steroids and base treatment on IMN will be assessed. We hope to explore whether SQG may improve the remission rate of primary membranous nephropathy in 6 months and 12 months, shorten the remission time and reduce side effects led by cyclical CTX/ steroids therapy.

### Objectives {7}

This study aims to evaluate the clinical efficacy and safety of SQG plus cyclical CTX/steroids and basic treatment in patients with IMN.

### Trial design {8}

This study is a multicenter, randomized, double-blind, placebo-controlled trial. The design of the trial was developed in accordance with Standard Protocol Items: Recommendations for Interventional Trials (SPIRIT) Guidelines [17].

Urinary protein excretion, serum albumin value and antibodies against the M-type phospholipase A2 receptor (PLA2Rab) titer are convenient for clinical evaluation. IMN patients with normal estimated glomerular filtration rate (eGFR) and proteinuria persistently exceed 3.5 g/d with conservative angiotensin converting enzyme inhibitor (ACEI)/angiotensin receptor blocker (ARB) therapy for at least 6 months following by serum albumin < 25 g/L or PLA2Rab > 50 RU/ml are assessed as in high risk. These patients will be screened. There 130 patients will be recruited for this study, and they were randomly divided into two groups according to the ratio of 1:1. The intervention group will receive SQG plus cyclical CTX/glucocorticoids therapy and basic treatment. The control group will receive cyclical CTX/glucocorticoids therapy plus basic treatment. Patients in intervention group will take SQG three times a day continuously for 6 months. Measurements of clinical efficacy and safety will be collected at baseline and every month until the end of the study (month 12).

**Fig. 1:**
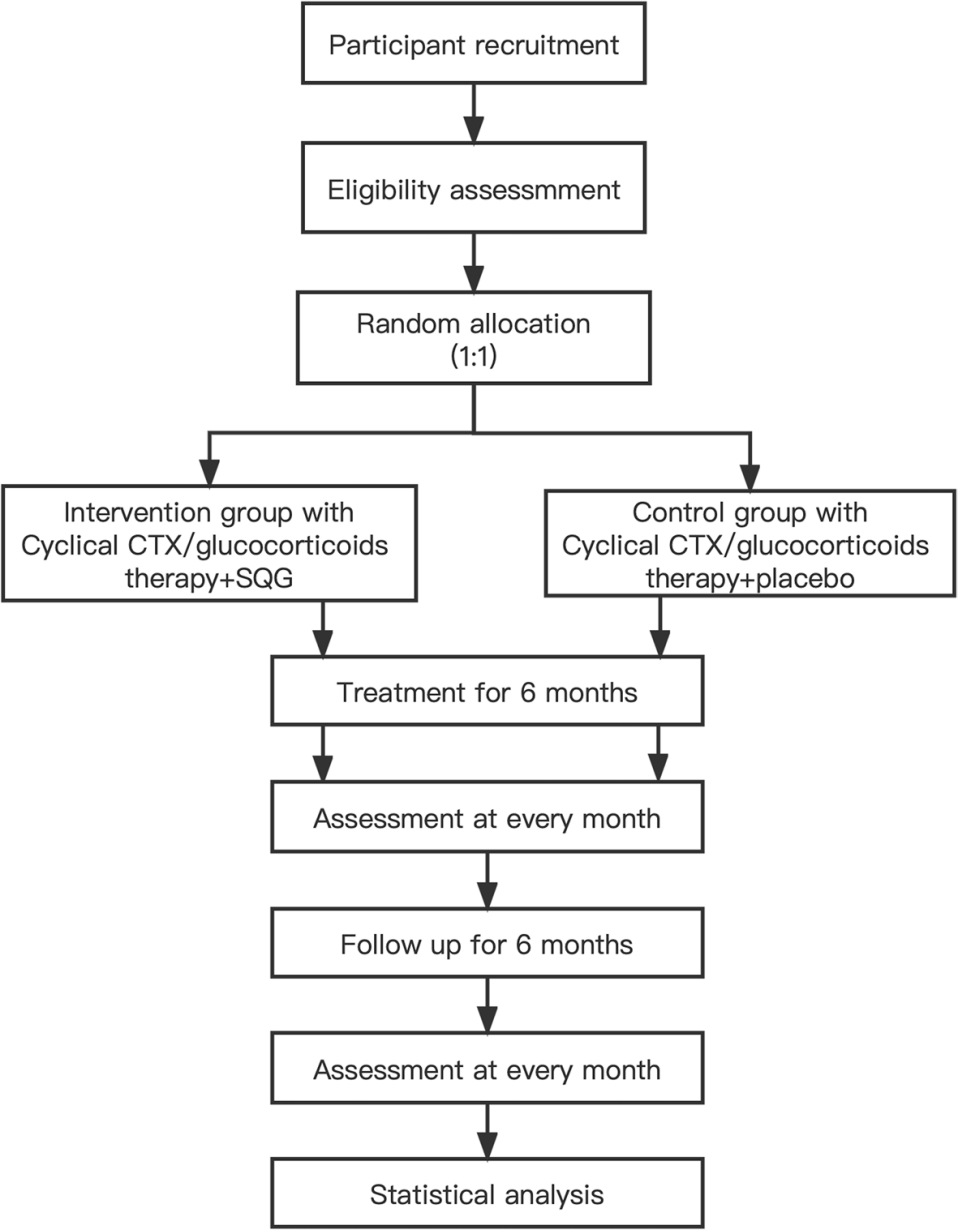
Flow chart

A flow chart of the study design is exhibited in Fig. 1

## Methods: Participants, interventions and outcomes

### Study setting {9}

The trial will be conducted in Guangdong Province Traditional Chinese Medical Hospital, Jiangsu Province Traditional Chinese Medical Hospital, the First Affiliated Hospital Guizhou University of Chinese Medicine, Shaanxi Province Traditional Chinese Medical Hospital, Shenzhen Traditional Chinese Medical Hospital.

### Eligibility criteria {10}

The inclusion criteria:

**——** Age ≥ 14 years old;
**——** Patients with determined diagnosis of IMN and need immunosuppressive therapy according to 2012 KDIGO guidelines;
**——** Patients agree to attend this study and sign the informed consent.

The exclusion criteria:

**——** Secondary membranous nephropathy, i.e., caused by hepatitis B, systematic lupus erythematodes (SLE), malignancy, etc.
**——** A known of contraindications and/or hypersensitivity to any ingredients of our research medications.
**——** Participating in another clinical interventional study.

### Who will take informed consent? {26a}

The researchers will receive patients and explain the contents of informed consent, including eligibility criteria, intervention, patient rights and interests, etc. If the patients have been evaluated as eligible, the written informed consent will be signed. The researchers will follow up with participants during the study.

### Additional consent provisions for collection and use of participant data and biological specimens {26b}

The participants will be informed that 5 ml of blood and 5 ml of urine of participants will be collected at base time, 6 months,12 months and the written informed consent will be signed.

## Interventions

### Explanation for the choice of comparators {6b}

Depending on KDIGO guidelines, high risk factors for progressive renal failure in IMN require immunosuppressive therapy. Compared with other immunosuppressants, CTX significantly improved the total remission and 24 h urinary total protein [18]. Intravenous cyclical CTX plus glucocorticoids treatment is available in most of the hospitals. However, its clinical application is limited due to obvious drug-related side effects such as infection, gastrointestinal symptoms and bone marrow suppression. The purpose of this study is to explore whether SQG could improve remission rates and reduce adverse effects from this standard therapy. Placebo and SQG are identical in appearance, including packaging, appearance, color and taste. So, cyclical CTX/ steroids + placebo are chosen as the comparator.

### Intervention description {11a}

SQG is a seven-flavor traditional Chinese medicine,including Tu Fu Ling (Rhizoma Smilacis Glabrae), Bai Zhu (Atractylodes macrocephala Koidz.), Huang Qi (Astragali Radix), Chan Hua (Isaria cicadae Miquel), E Zhu (Curcuma phaeocaulis Valeton), Chi Shao (Paeoniae Radix Rubra), San Qi (Panax notoginseng (Burkill) F. H. Chen ex C. H.). All herbs will be obtained from Guangdong Yifang Pharmaceutical Co., Ltd. (Foshan city, Guangdong, China). These herbs will be processed in granules form by hot water extracted, concentrated, spray-dried and packed in sealed sachets. The placebo is made from caramel (1.923%), sunset yellow (0.031%), sucrose octaacetate (0.023%), corn starch (10%), maltodextrin (86.1%), compound stabilizer 1(1.923%). The entire manufacturing process will comply with Chinese good manufacturing practices (GMP) by Guangdong Yifang Pharmaceutical Co., Ltd. (Foshan city, Guangdong, China). The SQG or placebo will be taken 1 sachet per time and 3 times per day for 6 months by participants.

Participants of both groups will be treated by cyclical CTX/steroids for 6 months. Both groups of patients received CTX + prednisone, CTX 500 mg/m^2^ intravenously every 4 weeks, a total of 6 pulses. Oral prednisone 1 mg/kg/d for 8 weeks, being reduced by 10 mg every 2 weeks to 30 mg/d and then tapering to by 5 mg every 2 weeks to 10 mg/d, and then being reduced by 2.5 mg every 2 weeks until drug withdrawal at the end of 6 months.

Participants of both groups will receive basic treatment and other concomitant medications for 6 months.

A ACEI/ARB drugs are the preferred antihypertensive medication after exclusion of contraindications, which can be used in combination with calcium ion antagonists or other antihypertensive drugs to control blood pressure to ≤ 140/90 mmHg, and > 90/60 mmHg, and maintain blood pressure stable.
B Symptomatic treatment for patients with hyperlipidemia, infection, edema, disturbance of electrolyte and acid–base metabolism, and hypercoagulability.
C The name, dosage, frequency and time of use of the drugs for comorbidities must be recorded in the study medical report.

### Criteria for discontinuing or modifying allocated interventions {11b}

**——**The eGFR decreased ≥ 40%from the baseline after treatment.
**——** Participants have been in ESRD, defined as, eGFR < 15 ml/min/1.73m^2^ and/or need renal replacement therapy.
**——** Occurrence of serious adverse events related to SQG plus cyclical CTX/ glucocorticoids therapy.
**——**Occurrence of drug allergy, as a result that the participants can not continue to complete the study.
**——**Occurrence of pregnancy during the treatment.
**——**Voluntary request for withdrawal by participants.

### Strategies to improve adherence to interventions {11c}

At the beginning of study,the participants will be informed that it will need enough time to attend the project. Researchers will provide them with convenient medical services. And the research team would regularly contact the patients by phone to reinforce the compliance with treatment and follow-up.

### Relevant concomitant care permitted or prohibited during the trial {11d}

Other types of glucocorticoids and immunosuppressants are prohibited. Tripterygium wilfordii polyglycosides tablets, Kunxian capsules, Huangkui capsules, Kunming Shanhaitang preparations or other Chinese patent medicines with similar efficacy and indications as SQG are also prohibited.

### Provisions for post-trial care {30}

During study, if there are side effects, we will continue to give appropriate treatment until the participants recover.

### Outcomes {12}

#### Primary outcomes

**——** Complete Remission (CR) rate—CR rate at the end of the 6th month and the 12th month.CR is defined as a value of 24 h urinary total protein lower than 0.3 g/d.
**——** Partial Remission (PR) rate—PR rate at the end of the 6th month and the 12th month.PR is defined as proteinuria > 0.3 g/d but < 3.5 g/d or 50% lower than baseline.

#### Secondary outcomes

**——** 24 h urinary total protein levels in urine at the time points of 3, 6, 9, 12 months.
**——** Urine Protein:Creatinine Ratio every month.
**——** eGFR every month.
**——** Serum anti-PLA2R levels at 3, 6, 9, 12 months respectively.
**——** Incidence of endpoint events. Endpoint events are defined as greater than or equal to 40%reduction in eGFR from baseline; or eGFR lower that 15 ml/min/1.73m^2^; or need for renal replacement therapy.
**——** Incidence of drug-related adverse events and severe adverse events.
**——** Changes in Dampness Syndrome Scale of Chinese Medicine [19] every month.

#### Safety outcomes

We will learn and collect any untoward medical occurrence that emerges or worsens relative to baseline by checking urine routine, blood routine, blood glucose, serum albumin, liver function, blood lipids, serum potassium, electrocardiogram, chest X-ray.

### Participant timeline {13}

The total course of our study is one year including 6 months treatment stage and subsequent 6 months follow-up stage. Participants will be evaluated at baseline and every month till end of the study. The time points of the trial are listed in Table 1.

**Table 1:** Schedule of enrollment, intervention, and assessment

Study Period													
	Baseline	Intervention phase						Follow-up phase					
Time point	0	M1	M2	M3	M4	M5	M6	M7	M8	M9	M10	M11	M12
Enrollment													
Eligibility screening	×												
Informed consent	×												
Randomization	×												
Interventions													
Cyclical CTX/glucocorticoids therapy + SQG	×	×	×	×	×	×	×						
Cyclical CTX/glucocorticoids therapy + placebo	×	×	×	×	×	×	×						
Assessments													
Demographic data	×												
Medical history	×												
Concomitant medication	×	×	×	×	×	×	×	×	×	×	×	×	×
Symptoms and signs	×	×	×	×	×	×	×	×	×	×	×	×	×
Physical examination	×	×	×	×	×	×	×	×	×	×	×	×	×
Routine blood and urine	×	×	×	×	×	×	×	×	×	×	×	×	×
24 h urinary total protein	×	×	×	×	×	×	×	×	×	×	×	×	×
Renal function test	×	×	×	×	×	×	×	×	×	×	×	×	×
Urine Protein:Creatinine Ratio	×	×	×	×	×	×	×	×	×	×	×	×	×
Serum anti-PLA2R levels	×			×			×			×			×
Liver function test	×	×	×	×	×	×	×	×	×	×	×	×	×
Serum albumin	×	×	×	×	×	×	×	×	×	×	×	×	×
Blood lipid	×	×	×	×	×	×	×	×	×	×	×	×	×
Blood glucose	×	×	×	×	×	×	×	×	×	×	×	×	×
Serum potassium	×	×	×	×	×	×	×	×	×	×	×	×	×
Electrocardiogram	×			×			×			×			×
Chest X-ray	×			×			×			×			×
Dampness Syndrome Scale of Chinese Medicine	×	×	×	×	×	×	×	×	×	×	×	×	×
Adverse events		×	×	×	×	×	×	×	×	×	×	×	×

*Abbreviations*: *M* month

### Sample size {14}

The TR rate of immunosuppressive therapy reported by different studies is inconsistent. To estimate the pooled TR rate of the control group, we conducted fixed-effects meta-analyses among data from five studies [20–24] by using the R software (version 4.0.2) plugin metaprop function. After calculation, the pooled TR rate of cyclical CTX/steroids regime is 56%(95%CI, 50.7–61.4) with a low heterogeneity (I^2^ = 0.000%, P < 0.000). From our previous study, the TR rate is 78.3%in the intervention group treated with SQG plus cyclical CTX/steroids for 6 months (unpublished data). PASS 11.0 was carried out to calculate the sample size with a 1:1 ratio based on the assumption of 56%vs 78.3% TR rate in the control vs intervention group. The study required an estimated 130 participants for an alpha of 0.05(single-tailed test), a power (1-β) of 0.81,and an allowed dropout rate of 15%.

### Recruitment {15}

The patients with proteinuria > 3.5 g/d, serum albumin < 25 g/L or PLA2Rab > 50 RU/mL will be recruited from Guangdong Province Traditional Chinese Medical Hospital, Jiangsu Province Traditional Chinese Medical Hospital, the First Affiliated Hospital Guizhou University of Chinese Medicine, Shaanxi Province Traditional Chinese Medical Hospital, Shenzhen Traditional Chinese Medical Hospital.

## Assignment of interventions: allocation

### Sequence generation {16a}

Methods of randomization have been introduced in our previous study [25, 26]. In briefly, all eligible subjects will be randomized 1:1. The PROC PLAN process of SAS V9.4 will be adopted to create a randomization number list. Randomized allocation will be conducted by the Key Unit of Methodology in Clinical Research (KUMCR).

### Concealment mechanism {16b}

The binding codes will be transmitted to each centres via a designated network randomization system to hide the allocation. Participants,researchers,statisticians are all blinded.

### Implementation {16c}

KUMCR will produce the allocation sequence. Researchers will recruit participants in every center. They will conduct assignment to interventions for each participants depending on the profile of the assigned group.

## Assignment of interventions: blinding

### Who will be blinded {17a}

The trial is a double-blind study, and all researchers, participants, and outcome assessors are blinded to the distribution of treatments.

### Procedure for unblinding if needed {17b}

KUMCR will label SQG and placebo by blind codes. KUMCR will keep all blind codes strictly confidential. Code breaks should only occur when participants experienced life-threatening adverse events. If unblinding is necessary, the researchers should contact KUMCR to get allocation group information corresponding to the blind code.

## Data collection and management

### Plans for assessment and collection of outcomes {18a}

The database of the trial is managed by Guangdong Province Hospital of Chinese Medicine. There are several dedicated clinical researchers in each sub-centre. The clinical research coordinators (CRCs) are responsible for collecting the data and then entering the data into a database for confirmation by the clinical research assistants (CRAs). Principal investigator (PI) will be responsible for quality management. Falsified data is prohibited.

### Plans to promote participant retention and complete follow-up {18b}

At the beginning of this study, the researchers will explain the sufficient follow-up information to participants. During the study, researchers will keep touch with participants to obtain follow-up data.

### Data management {19}

The members of data monitoring committee will regularly evaluate the quality of the data and safety events. The department of science research of Guangdong Provincial Hospital of Chinese Medicine will conduct the inspection for the study.

### Confidentiality {27}

All medical records and research materials will be kept strictly to avoid leakage. The information will be reserved by the researchers of Guangdong Provincial Hospital of Traditional Chinese Medicine and will never be transferred to other institutions.

### Plans for collection, laboratory evaluation and storage of biological specimens for genetic or molecular analysis in this trial/future use {33}

The 5 ml of blood and 5 ml of urine of participants will be collected at base time,6 months,12 months. After centrifuging,the serum and urine will be reserved at -80 °C refrigerator for molecular analysis in future use.

## Statistical methods

### Statistical methods for primary and secondary outcomes {20a}

The independent investigator in charge of statistical analysis will be unaware of the participants' identities and classification.

Continuous variables are described as mean, standard deviation, or maximum, minimum, median, and interquartile range, while categorical variables are reported as frequencies, percentage or constituent ratio.

Comparison of the measurement data, will be performed applying the paired t-test (or the paired signed rank-sum test Wilcoxon Signed Ranks Test) for comparison between groups. And two independent samples t-test (including 95%confidence interval calculation) will be used for comparison between groups, and rank-sum test (Wilcoxon W method) will be applied for non-normal distribution and unequal variance. If baselines between groups are inconsistent, analysis of covariance will be conducted.

To compare enumeration data, the composition ratio and rate of each index will be calculated, while the difference in the total effective rate between groups will be tested using the four-table test (or exact probability method) and the 2 × C table test will be used to compare the composition ratio between groups.

To analyze rank data, the paired signed rank-sum test (Wilcoxon Signed Ranks Test) will be used for the differences between before and after within the group, while the rank sum test (Mann–Whitney U method) for differences between groups.

The difference in the data of Dampness syndrome scale of Chinese Medicine between the two groups will be tested as rank data using Reliability Analysis, Factor Analysis, paired t test or paired signed rank sum test.

Center effect analysis will be conducted by Covariance analysis and Meta analysis for measurement data, and by Cochran-Mantel-Haenszel (CMH) test and Meta analysis (or Logsitic regression analysis) for enumeration data.

Analyze the influencing factors of curative effect will be performed by Unconditional logistic regression analysis.

The urinary protein remission rate and the median time to remission, the incidence of end-point events and the median time to the occurrence of end-point events in both groups will be compared using Survival analysis (Kaplan–Meier method). Cox regression analysis will be used to analyze the factors affecting the occurrence of end-point events in both groups.

### Interim analyses {21b}

There are no plans to conduct an interim analysis.

### Methods for additional analyses (e.g. subgroup analyses) {20b}

Methods for additional analyses is similar to statistical analysis of primary and secondary outcomes.

### Methods in analysis to handle protocol non-adherence and any statistical methods to handle missing data {20c}

The researcher will try to get in touch with the subjects to make up for the lack of information. The intention-to-treat analysis will be used to assessed the missing data.

### Plans to give access to the full protocol, participant level-data and statistical code {31c}

The full protocol, participant level-data and statistical code will be available for the authors as reasonably required during the study.

## Oversight and monitoring

### Composition of the coordinating centre and trial steering committee {5d}

The PI is responsible for monitoring the experiments. CRCs and CRAs conduct data coordination across centers. All of them are the members of the coordinating centre and trial steering committee, who will meet to discuss the problems of the trials implementation every three months.

### Composition of the data monitoring committee, its role and reporting structure {21a}

Data and safety monitoring committee, consisting of seven independent members, will be established to ensure data quality. They will be responsible for regularly evaluating the quality and safety of the data.

### Adverse event reporting and harms {22}

For any adverse events, investigators should report to their hospital and the Guangdong Provincial Hospital of Traditional Chinese Medicine Ethics Committee within 24 h. Adverse reactions should be noted and registered in the case report form during follow-up visits. The severity of the adverse reaction and the relationship to the related drug should be follow-up and investigated in detail. For all side effects, the researchers will immediately take the necessary steps to assure the safety of the trial participants. If the safety of trial participants can not be guaranteed, the study will be terminated prematurely.

### Frequency and plans for auditing trial conduct {23}

The research team meets every three months and conducts a sub-center field trip every six months, including trial implementation progress and data collection.

### Plans for communicating important protocol amendments to relevant parties (e.g. trial participants, ethical committees) {25}

Major changes to the research plan will be submitted to the Ethics Committee. At the same time, the online trial registration will be updated accordingly.

### Dissemination plans {31a}

Regardless of the outcome, the results of the study will be published in a peer-reviewed journal.

## Discussion

IMN grows up to be a global health problem and the number of IMN patients has increased rapidly in recent years [27]. The persistence and severity of IMN not only negatively affects the quality of life of the individual, but can also serve as a major burden to society. In the treatment of IMN, the treatment of moderate and severe IMN remains an important issue. KDIGO guidelines recommend that cyclical CTX/steroids be used for 6 months for MN patients to slow kidney failure and reduces the risk of developing end-stage renal disease [28]. After one year of treatment, the rate of patient's remissions ranged from 50 to 60% [29]. Nevertheless, severe infection, myelosuppression and gonadal suppression have greatly limited its clinical application [30, 31]. The frequent occurrence of serious adverse reactions is a major drawback of this treatment options. There is accumulated evidence showing that TCM can help relieve symptoms of nephrotic syndrome and improve patients' quality of life [32, 33]. SQG is a prescription for the treatment of kidney disease in traditional Chinese medicine. SQG is an extract from 7 herbs such as San Qi (Panax Notoginseng(Burk.)F.H.Chen Ex C.Chow), Huang Qi (Hedysarum Multijugum Maxim.), Tu Fu Ling (Rhizoma Smilacis Glabrae), Bai Zhu (Atractylodes macrocephala Koidz.), Chan Hua (Isaria cicadae Miquel), E Zhu (Curcuma phaeocaulis Valeton), Chi Shao (Paeoniae Radix Rubra.). Modern pharmacology has proved that herbs composing of SQG can be used individually for effective nephropathy treatment. Both San Qi and Huang Qi have promise for improvement renal function and optimization of the metabolism of serum lipids [34–36]. Tu Fu Ling water-extracts has significant positive effects on gentamicin-induced kidney injury as a result of its anti-oxidative properties [37]. Atractylenolide I, the active component of Bai Zhu extracts could represses the myofibroblastic phenotype and fibrosis development in UUO kidneys [38]. A study has proved that E Zhu could induce apoptosis in the A498 renal carcinoma cell line as a result of its antitumorogenic properties [39]. Paeoniflorin, the main constituents of Chi Shao extracts, could significantly attenuate the functional and histological damage to diabetic mice kidney [40]. The beneficial effects of SQG might come from the combination of multiple components from the different herbs.

SQG is derived from Sanqi formula which containing San Qi and Huang Qi. In the past 20 years, our research team has made continuous efforts to reveal the efficacy, safety, and pharmacological mechanism of Sanqi formula. Our previous basic studies have also shown that Sanqi formula could effectively help rat model of membranous nephropathy via reducing proteinuria, increasing serum albumin, ameliorating renal damage which is associated with the suppression of nuclear factor-kappa B (NF-κB) [41]. Besides, our studies reveal that Sanqi Oral Liquid protect podocyte as evidenced by effectively reducing podocytes apoptosis which is associated with nuclear factor erythroid 2-related factor 2 (Nrf2)/heme oxygenase-1 (HO-1) signaling pathway [42]. Our another experimental studies based on rat model of renal ischemia/reperfusion (I/R) injury demonstrated that Sanqi Oral Liquid has a significant effect on the immune system and exerts renoprotective effects by regulating apoptosis and autophagy, which may be linked to extracellular signal-regulated kinase (ERK)/mammalian target of rapamycin (mTOR) pathways [43].

TCM is an alternative therapy to treat IMN, however, high-quality evidence-based medicine evidence is insufficient. This clinical study is a rigorously designed, multicenter, randomized, double-blind, placebo-controlled trial to assess the efficacy and safety of SQG combined with cyclical CTX/steroids in the treatment of IMN. This trial is the first clinical study of SQG plus first-line drugs to treat IMN. The results of this trial will provide a high-quality evidence-based medicine evidence for the clinical application of SQG and lead to multiple drug options for patients with IMN.

## Trial status

At the time of manuscript submission,the trial is in the phase of recruiting participants. We recruit participants from July 20, 2022 to June 1, 2026.

## Data Availability

The data used and analyzed in the research process will be provided by the relevant authors as reasonably required.

## Abbreviations

IMN: Idiopathic membranous nephropathy
MPR: Modified Ponticelli Regimens
SQG: Sanqi Qushi Granule
CTX: Cyclophosphamide
PLA2R: M-type phospholipase A2 receptor
ESRD: End-stage renal disease
KDIGO: Kidney Disease Improving Global Outcomes
TCM: Traditional Chinese Medicine
SPIRIT: Standard Protocol Items: Recommendations for Interventional Trials
PLA2Rab: Antibodies against the M-type phospholipase A2 receptor
eGFR: Estimated glomerular filtration rate
ACEI: Angiotensin converting enzyme inhibitor
ARB: Angiotensin receptor blocker
SLE: Systemic lupus erythematodes
h: Hour
GMP: Chinese good manufacturing practices
CR: Complete Remission
PR: Partial Remission
KUMCR: Key Unit of Methodology in Clinical Research
CRCs: Clinical research coordinators
CRAs: Clinical research assistants
PI: Principal Investigator
M: Month
CMH: Cochran-Mantel-Haenszel
NF-ĸB: Nuclear factor-kappa B
Nrf2: Nuclear factor erythroid 2-related factor 2
HO-1: Heme oxygenase-1
I/R: Ischemia/reperfusion
ERK: Extracellular signal-regulated kinase
mTOR: Mammalian target of rapamycin
LAR: Legally Authorized Representative
